# Supplementary material for: Engineered Methylobacterium extorquens grows well on methoxylated aromatics due to its formaldehyde metabolism and stress response
Source: mSphere. 2025 Jul 31;10(8):e00171-25. doi: 10.1128/msphere.00171-25 (PMC12379598; doi:10.1128/msphere.00171-25)
Supplement: Fig. S1 to S3 — Additional growth experiments and plasmid map. [file msphere.00171-25-s0001.pdf]

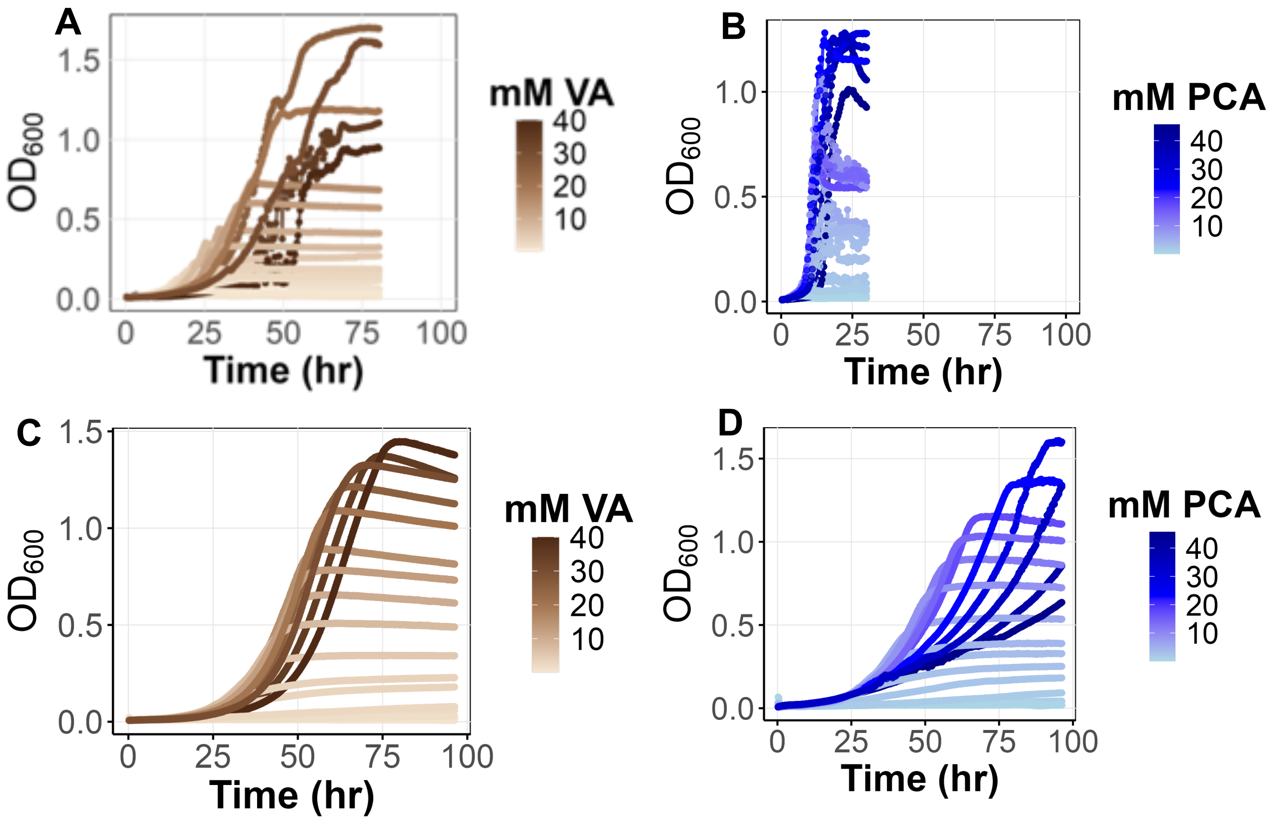

Figure S1: Comparative growth of *M. nodulans* and engineered *M. extorquens* on VA and PCA. Growth curves of *M. nodulans* (A, B) and engineered *M. extorquens* (C, D) with increasing concentrations of VA (A, C, red-yellow gradient) and PCA (B, D blue gradient).

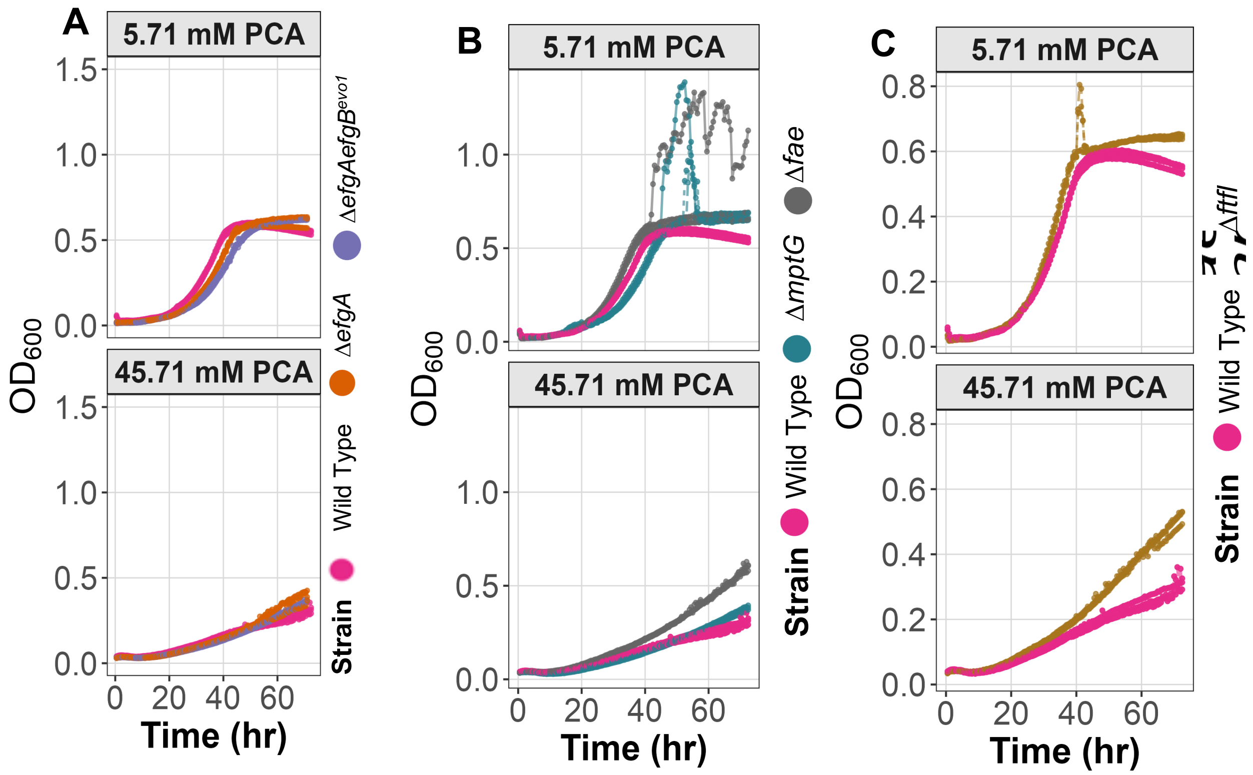

Figure S2: Growth curves of different strains on PCA. Strains with formaldehyde-evolved alleles (A), oxidation mutants (B), and assimilation mutants (C) on PCA. None of the VA defects or advantages noted for these strains carried over to PCA, indicating their specificity to VA. Some strains exhibited new phenotypes on PCA, such as an advantage for the Δ*fae* strain, for reasons which we do not yet understand.

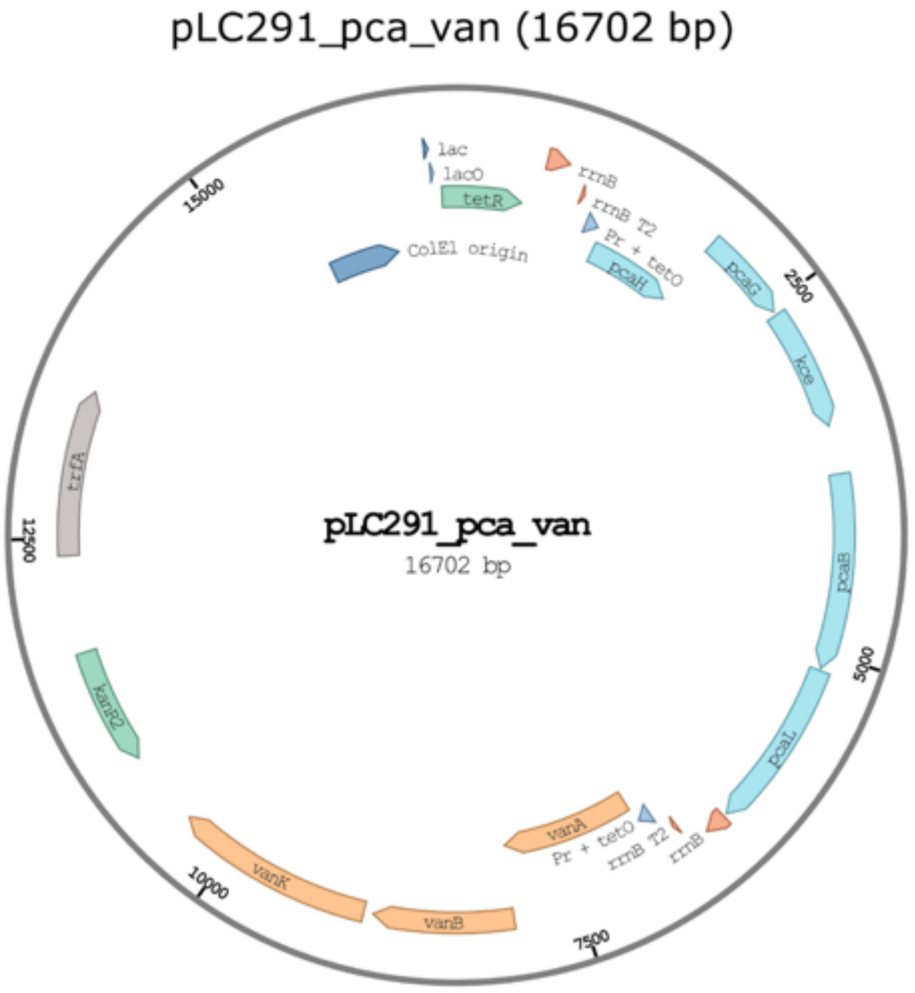

Figure S3: Plasmid-map for pLC291-*pca-van*.
